# Childhood Idiopathic Nephrotic Syndrome: Does the Initial Steroid Treatment Modify the Outcome? A Multicentre, Prospective Cohort Study

**DOI:** 10.3389/fped.2021.627636

**Published:** 2021-07-08

**Authors:** Andrea Pasini, Cristina Bertulli, Luca Casadio, Ciro Corrado, Alberto Edefonti, GianMarco Ghiggeri, Luciana Ghio, Mario Giordano, Claudio La Scola, Cristina Malaventura, Silvio Maringhini, Antonio P. Mastrangelo, Marco Materassi, Francesca Mencarelli, Giovanni Messina, Elena Monti, William Morello, Giuseppe Puccio, Paola Romagnani, Giovanni Montini, the NefroKid Study Group

**Affiliations:** ^1^Nephrology and Dialysis Unit, Department of Pediatrics, IRCCS Azienda Ospedaliero-Universitaria di Bologna, Bologna, Italy; ^2^Unità Operativa Complessa of Paediatrics and Neonatology, Local Health Authority of Romagna, Ravenna, Italy; ^3^Pediatric Nephrology Unit, Children's Hospital “G. Di Cristina”, A.R.N.A.S. “Civico”, Palermo, Italy; ^4^Pediatric Nephrology, Dialysis and Transplant Unit, Fondazione Ca' Granda Istituto di Ricovero e Cura a Carattere Scientifico Ospedale Maggiore Policlinico, Milan, Italy; ^5^Division of Nephrology, Dialysis, Transplantation, Laboratory on Pathophysiology of Uremia, Istituto G. Gaslini, Genoa, Italy; ^6^Nephrology Unit, Giovanni XXIII Children's Hospital, Bari, Italy; ^7^Section of Pediatrics, Department of Medical Sciences, University of Ferrara, Ferrara, Italy; ^8^Nephrology and Dialysis Unit, Meyer Children's Hospital, Florence, Italy; ^9^Specialty School of Paediatrics - Alma Mater Studiorum, Università di Bologna, Bologna, Italy; ^10^Retired, Palermo, Italy; ^11^Giuliana and Bernardo Caprotti Chair of Pediatrics, Department of Clinical Sciences and Community Health, University of Milano, Milan, Italy

**Keywords:** childhood idiopathic nephrotic syndrome, steroid treatment, frequent relapsers, steroid dependency, prognostic factors, age at onset, total serum protein

## Abstract

**Background:** A great majority of children with idiopathic nephrotic syndrome will relapse after successful treatment of the initial episode. The possibility that different steroid dosing regimens at onset, adjusted for risk factors, can reduce the rate of relapse represents an interesting option to investigate.

**Objectives:** To evaluate the effect of the initial steroid regimen, adjusted for time to remission (TTR), on the frequency of relapses and steroid dependence, and to verify the influence of prognostic factors on disease course.

**Methods:** A multicentre, prospective, cohort study. Children with nephrotic syndrome, with TTR ≤ 10 days (Group A), were given a 20-week prednisone regimen (2,828 mg/m^2^) and those with a TTR >10 days, a 22-week regimen (3,668 mg/m^2^) (Group B). Previously published retrospective data from the same centers were also evaluated. Main outcomes were: relapse rate, number of frequent relapsers + steroid dependent children and total prednisone dose after induction.

**Results:** 143 children were enrolled. Rate of relapsed subjects (77 vs. 79%) and frequent relapsers + steroid dependent subjects (40 vs. 53%) did not differ between Groups A and B, or between the retrospective and prospective cohorts. The cumulative prednisone dose taken after the induction treatment was similar in both groups and in the retrospective and prospective cohorts. TTR was not associated with relapse risk. Age at onset and total serum protein were significantly lower in relapsing patients. At ROC analysis, the best cut-off was 5.3 years for age at onset and 4.2 g/dL for total serum protein. According to these cut-offs, older children with higher total serum protein had a higher relapse free survival rate (58%) than younger children with lower total serum protein (17%).

**Conclusions:** TTR was not found to be a prognostic factor of relapse; because of this, different steroid regimens, adjusted for TTR, did not modify the relapse rate in any relevant measure. Conversely, younger age and low total serum protein were independent predictors of relapse risk, however this outcome was not modified by higher prednisone regimens.

**Clinical Trial Registration:**https://www.ClinicalTrials.gov/, identifier: NCT01386957 (www.nefrokid.it).

## Introduction

Steroid therapy is the first-line treatment for idiopathic nephrotic syndrome (INS), inducing remission in 80–90% of children (1–3). However, 75–80% of responders will relapse, and 40–50% will show frequent relapses or steroid dependence (4–9). No consensus exists regarding optimal dosing or treatment duration (10–16). In 2000, a Cochrane meta-analysis showed that 3 months of steroid treatment resulted in a lower relapse rate at 12 and 24 months, with an increased benefit being demonstrated for up to 7 months of treatment (17). While new trials focused on modifying the clinical evolution of INS, many study groups began looking for prognostic factors of relapse and steroid dependence [early age at onset (18–21), male gender (21), intrauterine growth retardation (22), and time to remission (23–25)]. Vivarelli et al. identified time to remission (TTR) >14 days as a main prognostic factor for relapse, with all subjects relapsing within 3 months, while 20% of subjects with a TTR ≤ 7 days were still in remission at 18 months.

On these bases, we designed an epidemiological, observational, prospective, multicenter study to evaluate the role of the initial steroid regimen as regards relapse occurrence and steroid dependence, and to verify the influence of potential prognostic factors on disease course (23–25).

Moreover, we took the clinical decision to differentiate the steroid regimen on the basis of TTR (≤ 10 or >10 days), with the aim of protecting later responders from the higher risk of relapse, as reported by some authors (23–25).

## Methods

This is an epidemiological, multicenter, prospective cohort study involving 49 Italian pediatric units, performed between 2011 and 2016 (ClinicalTrials.gov Id: NCT01386957). An additional cohort, compiled using retrospective data from children treated at the same centers, was used for further analyses (13). Ethics Committee approval from the participating hospitals and written informed consent were obtained.

### Diagnosis

All children with a first episode of INS, defined as proteinuria >40 mg/m^2^/h or urine protein/creatinine ratio (uPr/uCr) >2 mg/mg and albuminemia <2.5 g/dL, were enrolled. Inclusion criteria were age at onset >6 months and <18 years and a diagnosis of INS. Exclusion criteria were congenital and secondary forms of nephrotic syndrome and steroid resistance. During the treatment protocol, dipstick urinalysis was performed daily, in order to identify TTR (first day of negative/trace dipstick).Relapse was defined as 3 days of dipstick ≥2+, confirmed by uPr/uCr >2 mg/mg. Time to relapse was defined as the time elapsed since the start of treatment to the first relapse. At the end of the 24-month follow-up period, patients were classified as non-relapser (NR), infrequent relapser (IR), frequent relapser (FR), or steroid-dependent (SD) according to standard definitions (26). Non-relapsers and infrequent relapsers (NR + IR) and frequent relapsers and steroid-dependent (FR + SD) subjects were grouped and compared.

### Therapy Protocol

Subjects with a TTR ≤ 10 days (Group A) received prednisone 60 mg/m^2^/day for 4 weeks, those with a TTR >10 days (Group B) for 6 weeks. Patients not achieving remission within 6 weeks received 3 alternate-day pulses of iv methylprednisolone (1 g/1.73 m^2^, max 1g), followed by alternate-day prednisone at 40 mg/m^2^. Those who had not achieved remission after a further 2 weeks were classified as steroid-resistant (SR). Steroid tapering was identical for all patients: 4 weeks of alternate-day prednisone (40 mg/m^2^), followed by 14 weeks of tapering. The first episode cumulative prednisone dose was 2,828 mg/m^2^ (20 weeks) in Group A, and 3,668 mg/m^2^ (22 weeks) in Group B. First relapses were treated with prednisone 60 mg/m^2^/day, until proteinuria was negative for 5 consecutive days, then a single alternate-day 40 mg/m^2^ dose (4 weeks). Subsequent relapses were treated according to each individual center's relapse protocol.

### Clinical and Laboratory Data

Height, weight, body mass index, systolic and diastolic blood pressure (SDS), complete blood count, urea, creatinine, uricemia, serum protein electrophoresis, albumin, total cholesterol, triglycerides, electrolytes, urinalysis, 24-h proteinuria, or uPr/uCr were recorded in an online database (www.nefrokid.it) at diagnosis, 12 and 24 months. Total number of relapses, time to relapse, total steroid dose at 12 and 24 months, and the use of other immunosuppressors were also recorded.

### Additional Retrospective Cohort

We acknowledged that the original design of the prospective study was somewhat flawed. Therefore, we decided to get a wider perspective in order to draw more reliable conclusions (see Discussion). To that purpose, a previously published retrospective study of 144 INS children diagnosed between January 2007 and December 2009 and followed up for 24 months was used as an additional cohort for further analyses (13). Inclusion criteria were identical, while steroid treatment was not standardized. Steroid induction dose was 2,013 ± 617 mg/m^2^, with a 5 (2.5–8) week duration. The first episode mean cumulative prednisone dose was 3,582 ± 881 mg/m^2^, ranging from 1,904 to 6,035 mg/m^2^, with a 21 (9–48) week duration.

#### Study Aims

(a) To evaluate and compare the clinical course of patients (Group A vs. Group B and prospective vs. retrospective cohort) at 24 months.
(b) To evaluate the prognostic relevance of the following factors: age and laboratory data at onset, TTR in continuous form, total prednisone induction dose (4 weeks of daily prednisone vs. 6 weeks).

#### Outcomes

(a) Relapse free survival, percentage of patients with at least one relapse at 24 months, number of relapses per patient and time to first relapse.
(b) Steroid sensitivity (prevalence of FR + SD subjects) at 24 months.
(c) Cumulative post-induction prednisone dose (cumulative prednisone dose administered from induction to 24 months).

### Statistical Analysis

Statistical analysis was performed using the open source software R (27). The Chi-Square test of independence was used to analyze the association between categorical variables. Non-parametric tests (Wilcoxon, Kruskal-Wallis) were used to compare the distribution of a continuous variable in two or more different groups.

Linear regression models were used to analyse the association between continuous variables and the Kaplan Meier estimator to build relapse free survival curves. Cox hazard ratio models were used to evaluate significance of prognostic factors in relation to survival curves and for multivariate analysis.

## Results

### Clinical Course

#### Prospective Cohort

**Figure 1:**
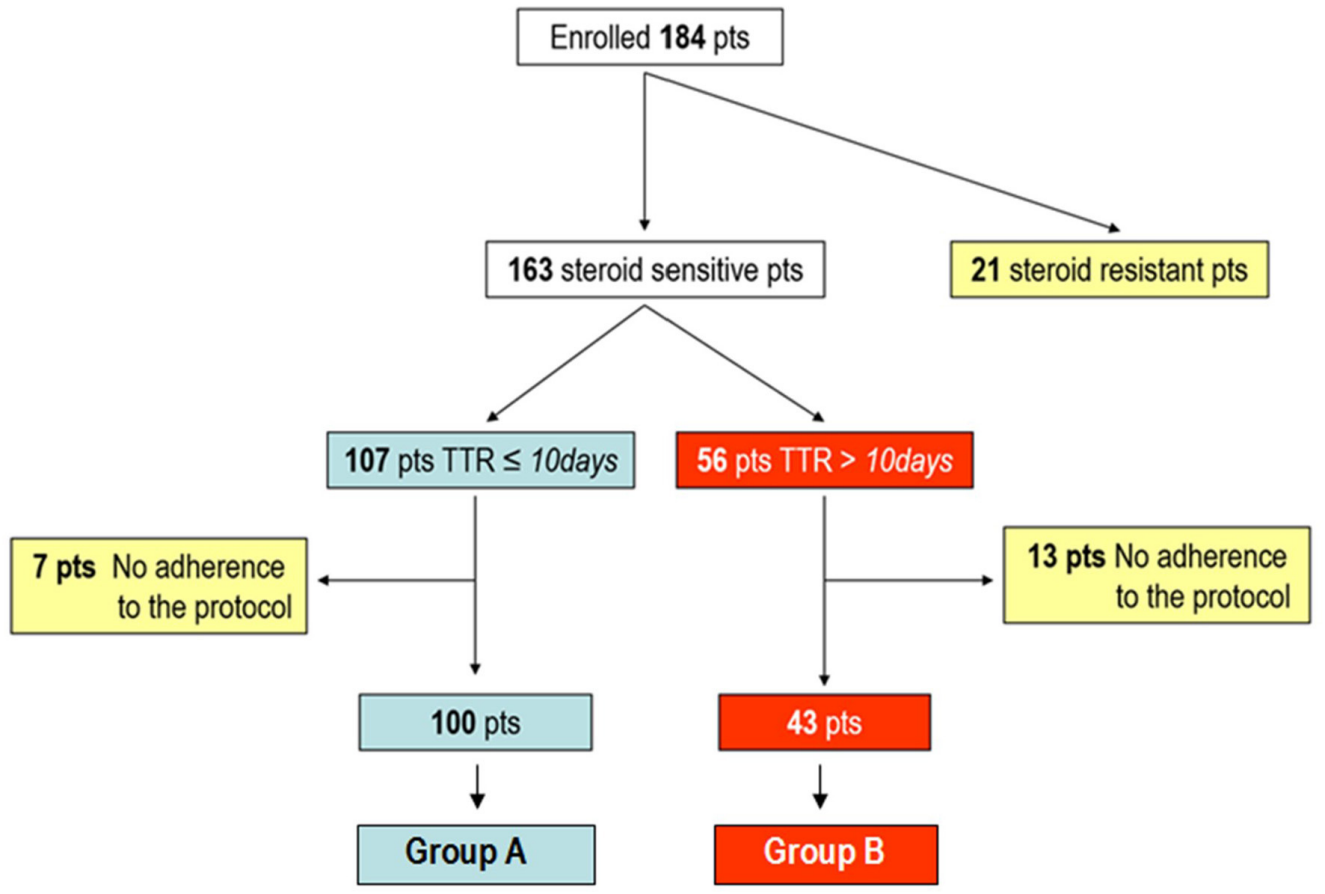
Study flow chart.

One hundered and eighty-four children (median age at diagnosis: 3.9, range 0.6–17 years; Male:Female 1.9:1) with INS were enrolled. One hundred and sixty-three (89%) were steroid-sensitive (SS), 21 (11%) were SR and were excluded alongside an additional 20 patients due to non-compliance. The final cohort comprised 143 subjects. Among them, 100 (70%) had a TTR ≤ 10 days and were administered the 4-week induction regimen (Group A), whilst the 43 with a later TTR were given the 6-week induction regimen (Group B) (Figure 1).

#### Retrospective Cohort

One hundred forty-four steroid sensitive patients from a retrospective study (13), followed-up for 24 months, were evaluated. Steroid induction dose was 2,013 ± 617 mg/m^2^, with a 5 (2.5–8) week duration.

#### Data at Onset

Clinical characteristics and laboratory data are shown in Table 1. The two cohorts were similar, except for higher serum creatinine and lower serum albumin values in the retrospective cohort. Groups A and B were also similar, except for age at onset, which was significantly lower in Group B, while urea, uricemia, total cholesterol, Na, K, P, and PrU/CrU were significantly higher in Group B.

**Table 1:** Comparison of clinical and laboratory data at onset: Group A vs. Group B and prospective vs. retrospective cohort.

	**Prospective cohort**			**Restrospective cohort**	***p*****-value**			
	**Total**	**Group A**	**Group B**	**Total**	**Group A vs. Group B**	**Adjusted (Holm's method)**	**Prospective vs. retrospective**	**Adjusted (Holm's method)**
	**(143 pts)**	**(100 pts)**	**(43 pts)**	**(144 pts)**				
**Clinical data**								
Age (years)	4.55 ± 2.7	4.73 ± 2.53	4.14 ± 3.06	4.75 ± 3.06	0.01	0,21	0.885	1
Sex (Male)	93 (65.0%)	65 (65.0%)	28 (65.1%)	96 (66.7%)	0.989	1	0.771	1
Height (SDS)	−0.04 ± 0.87	−0.08 ± 0.81	0.05 ± 1.01	0.02 ± 1.00	0.412	1	0.481	1
Weight (SDS)	0.46 ± 0.89	0.4 ± 0.91	0.64 ± 0.84	0.55 ± 1.03	0.082	1	0.171	1
BMI (SDS)	0.65 ± 0.93	0.60 ± 0.92	0.79 ± 0.95	0.72 ± 0.89	0.207	1	0.541	1
sBP (SDS)	1.07 ± 1.12	1.01 ± 1.05	1.23 ± 1.27	0.86 ± 1.14	0.294	1	0.083	1
dBP (SDS)	1.34 ± 0.88	1.28 ± 0.76	1.5 ± 1.12	1.13 ± 0.91	0.529	1	0.150	1
SGA	16/143	10/100 (10%)	6/43 (13.8%)		0.491	1		
**Laboratory data**								
Hemoglobin (g/dL)	13.0 ± 1.2	13.08 ± 1.09	12.97 ± 1.34	13.3 ± 1.2	0.323	1	0.104	1
Urea (mg/dL)	29.3 ± 16.5	26.66 ± 13.25	35.26 ± 21.07	28.6 ± 13.2	0.01	0,21	0.936	1
Creatinine (mg/dl)	0.30 ± 0.15	0.29 ± 0.12	0.34 ± 0.21	0.35 ± 0.15	0.653	1	0.001	0.022
Uricemia (mg/dL)	4.23 ± 0.99	4.04 ± 0.93	4.65 ± 1.01	4.13 ± 1.09	0.004	0,088	0.546	1
Total proteins (g/dL)	4.23 ± 0.58	4.26 ± 0.59	4.18 ± 0.58	4.21 ± 0.67	0.323	1	0.734	1
Albumin (g/dL)	1.63 ± 0.50	1.64 ± 0.52	1.60 ± 0.46	1.40 ± 0.40	0.955	1	0.001	0.022
Tot. cholesterol (mg/dL)	403.7 ± 108.1	395.7 ± 108.05	421.09 ± 107.49	400.1 ± 102.1	0.049	0,784	0.665	1
Triglycerides (mg/dL)	197.20 ± 110.2	184.2 ± 92.12	225.86 ± 139.03	217.62 ± 138.5	0.074	1	0.331	1
Na (mmol/L)	136.8 ± 3.5	137.25 ± 3.54	135.84 ± 3.11	136.2 ± 3.4	0.03	0,522	0.240	1
K (mmol/L)	4.47 ± 0.50	4.42 ± 0.43	4.61 ± 0.52	4.51 ± 0.56	0.02	0,38	0.921	1
Ca (mg/dL)	8.18 ± 0.55	8.15 ± 0.61	8.24 ± 0.42	8.18 ± 0.70	0.439	1	0.652	1
P (mg/dL)	4.93 ± 0.82	4.84 ± 0.81	5.12 ± 0.82	5.08 ± 1.09	0.029	0,522	0.287	1
uPr/uCr (mg/mg)	13.39 ± 10.7	11.74 ± 9.41	17.44 ± 12.6	10.32 ± 7.22	0.002	0.046	0.056	1
Proteinuria (g/L)	9.32 ± 9.13	8.29 ± 8.27	11.91 ± 10.71	8.05 ± 8.17	0.088	1	0.230	1
Microhematuria	58 (40.6%)	37 (37.0%)	22 (51.2%)	59 (41.0%)	0.114	1	0.943	1
**Steroid response**								
TTR (days)	10.3 ± 7.8			14.8 ± 12.4			0.00025	0.00575

*Mean ± SD for numerical variables, number (%) for categorical variables. BMI, body mass index; sBP, systolic blood pressure; dBP, dyastolic blood pressure; SGA, small for gestational age. The red color values represent the significant values*.

The median TTR was 8 (1–47) days in the prospective cohort, with 156 (96%) patients achieving remission within 4 weeks, and 7 (4%) in more than 4 weeks (3 after iv methylprednisolone boluses). The median TTR was significantly longer (11; range 3–77 days) in the retrospective cohort. The mean total induction prednisone dose was 1,685 ± 107 mg/m^2^ in Group A, 2,648 ± 426 in Group B, and 2,013 ± 617 in the retrospective cohort.

#### Relapses

**Figure 2:**
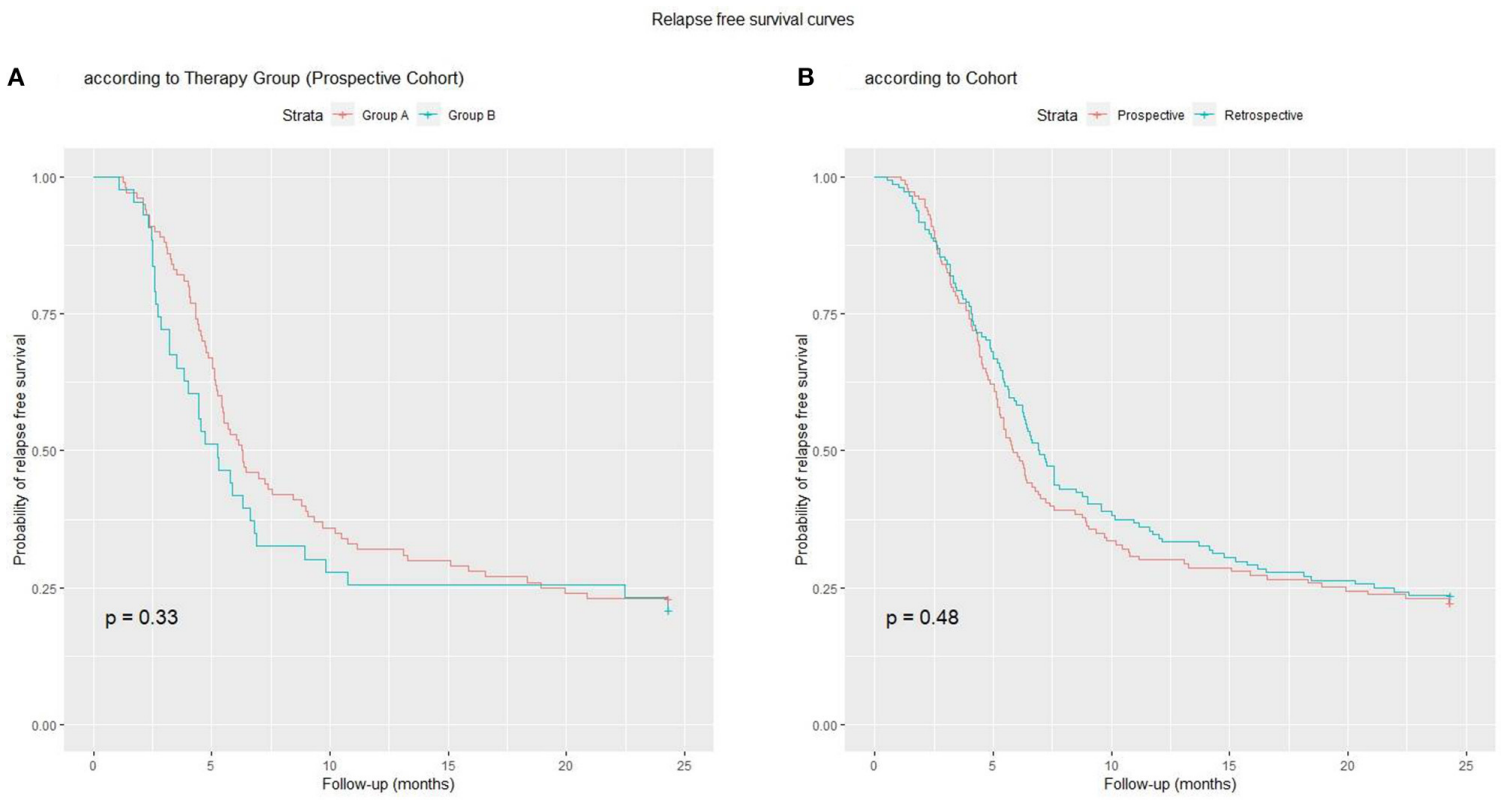
Relapse-free survival curves: **(A)** Group A vs. Group B; **(B)** Prospective vs. Retrospective Cohort.

In the prospective cohort, 111 (78%) subjects relapsed after a mean time of 188 ± 140 days from the start of treatment. At 12 months, 100 subjects (70%) had relapsed. There were a total of 268 relapses (182 in Group A, 86 in Group B), ranging from 1 (42 patients) to 7 (1 patient) per patient. The mean number of relapses per patient (1.8 vs. 2; *p* = 0.43), and the percentage of patients who had relapsed at 12 (68 vs. 74%; *p* = 0.44) and 24 months (77 vs. 79%; *p* = 0.78) did not differ between Groups A and B. The relapse rate in the retrospective vs. the prospective cohort (77 vs. 78% at 24 months, *p* = 0.91) and the number of relapses per patient (2.1 vs. 1.9, *p* = 0.44) did not differ. The time to first relapse was longer in Group A vs. Group B (mean = 205.6 vs. 166.8 days; *p* = 0.05). The relapse-free survival curve up to the end of follow-up did not differ between Groups A and B (p=0.30), or between the two cohorts (*p* = 0.50; Figures 2A,B).

#### Steroid Sensitivity

In the prospective cohort, 32 children (22%) were NR, 48 (34%) IR, 5 (3%) FR, 58 (41%) SD. The prevalence of FR + SD subjects was similar in Groups A and B (40 vs. 53%, *p* = 0.14) and in the two cohorts (43 vs. 44%, *p* = 0.8).

#### Cumulative Post-induction Prednisone Doses

**Figure 3:**
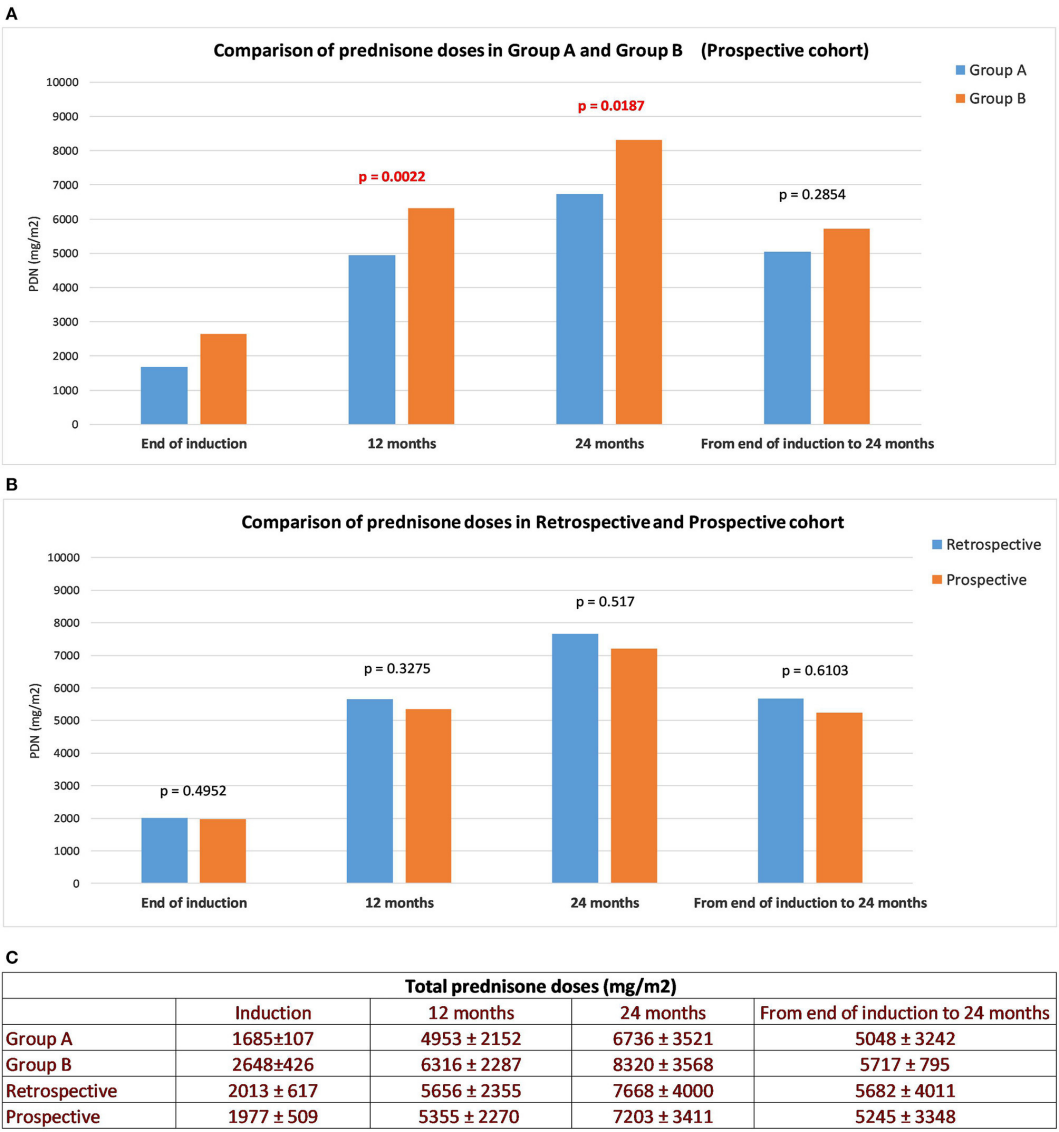
Cumulative prednisone doses. Total daily PRED dose, cumulative PRED dose at 12, 24 months and from the end of induction to the end of follow-up: Group A vs. B **(A)**, prospective vs. retrospective cohort **(B)**. All values are means ± SD (mg/m^2^) **(C)**.

The Group A induction dose was lower per protocol (4 vs. 6 weeks) and the cumulative dose remained significantly lower at 12 and 24 months (*p* = 0.002 and *p* = 0.018, respectively), compared to Group B. Therefore, the cumulative post-induction prednisone dose did not differ significantly (*p* = 0.28; Figure 3A). Steroid sparing agents were utilized more frequently in Group B than in Group A (42 vs. 25%, *p* = 0.04). When comparing the retrospective and prospective cohorts, the mean total induction prednisone dose (2013 vs. 1977, *p* = 0.49), the mean cumulative prednisone dose at 12 (5,656 vs. 5,355, *p* = 0.3) and 24 months (7,668 vs. 7,203, *p* = 0.5) and the mean cumulative prednisone dose administered from induction to 24 months (5,682 vs. 5,245, *p* = 0.6) did not differ (Figure 3B).

### Prognostic Factors

The significance of prognostic factors for the whole population (prospective + retrospective cohort) is shown in Table 2, and separately for the prospective and retrospective cohorts in Supplementary Tables 1, 2, respectively.

**Table 2:** Prognostic factors evaluated in the whole population according to the different outcomes.

	**Relapse**	**Time to relapse**	***N*. of relapses**	**Prevalence of FR-SD**	**PDN after remission**	**Relapse-free survival**	**Relapse-free survival adjusted (Holm's method)**
**Whole population**							
Age at onset (years)	0.00002	0.0006	0.0009	0.00005	0.046	0.0000001	0.000002
TTR (days)	0.088	NS	NS	0.003	NS	NS	NS
Prednisone dose in induction (mg/m2)	NS	NS	NS	NS	NS	NS	NS
Hemoglobin (g/dL)	NS	NS	NS	NS	NS	NS	NS
Urea (mg/dL)	NS	NS	NS	NS	0.005	NS	NS
Creatinine (mg/dl)	0.016	NS	0.012	0.042	0.017	0.0037	0.056
Uricemia (mg/dL)	NS	NS	NS	0.07	NS	0.096	NS
Total proteins (g/dL)	0.0003	0.009	0.005	0.019	0.00002	0.00001	0.00019
Albumin (g/dL)	0.058	0.004	NS	0.086	0.025	0.0009	0.0162
Alpha 2 globulins (g/L)	NS	NS	NS	NS	NS	NS	NS
Gamma globulins (g/L)	NS	NS	NS	NS	NS	0.068	NS
Tot. cholesterol (mg/dL)	0.049	NS	NS	NS	0.09	NS	NS
Triglycerides (mg/dL)	0.08	NS	NS	0.056	NS	0.059	NS
Na (mmol/L)	0.06	0.02	NS	NS	0.06	0.039	NS
K (mmol/L)	NS	NS	NS	NS	NS	NS	NS
Ca (mg/dL)	NS	NS	NS	NS	0.014	NS	NS
P (mg/dL)	NS	NS	NS	NS	NS	NS	NS
uPr/uCr (mg/mg)	0.039	0.016	NS	0.00016	0.026	0.001	0.017
Proteinuria (g/L)	NS	0.007	0.046	0.002	0.0006	0.001	0.017
Microhematuria	NS	NS	NS	NS	NS	NS	NS

*p-values are in red if <0.05, in green if ≥0.05, and <0.10. All p > 0.10 are labeled as NS (not significant)*.

#### Age at Onset

Age at onset was significantly associated with all outcomes in the whole population (Table 2) and in the prospective cohort. In the retrospective cohort, only time to relapse and prednisone dose were not significantly associated with age.

#### TTR

In the whole population, TTR in continuous form was only significantly associated with a higher prevalence of FR-SD patients (Table 2). This association held true in the retrospective cohort, where a weak association with a higher relapse rate was also seen (*p* = 0.02). No associations were observed in the prospective cohort (Supplementary Tables 1, 2).

**Figure 4:**
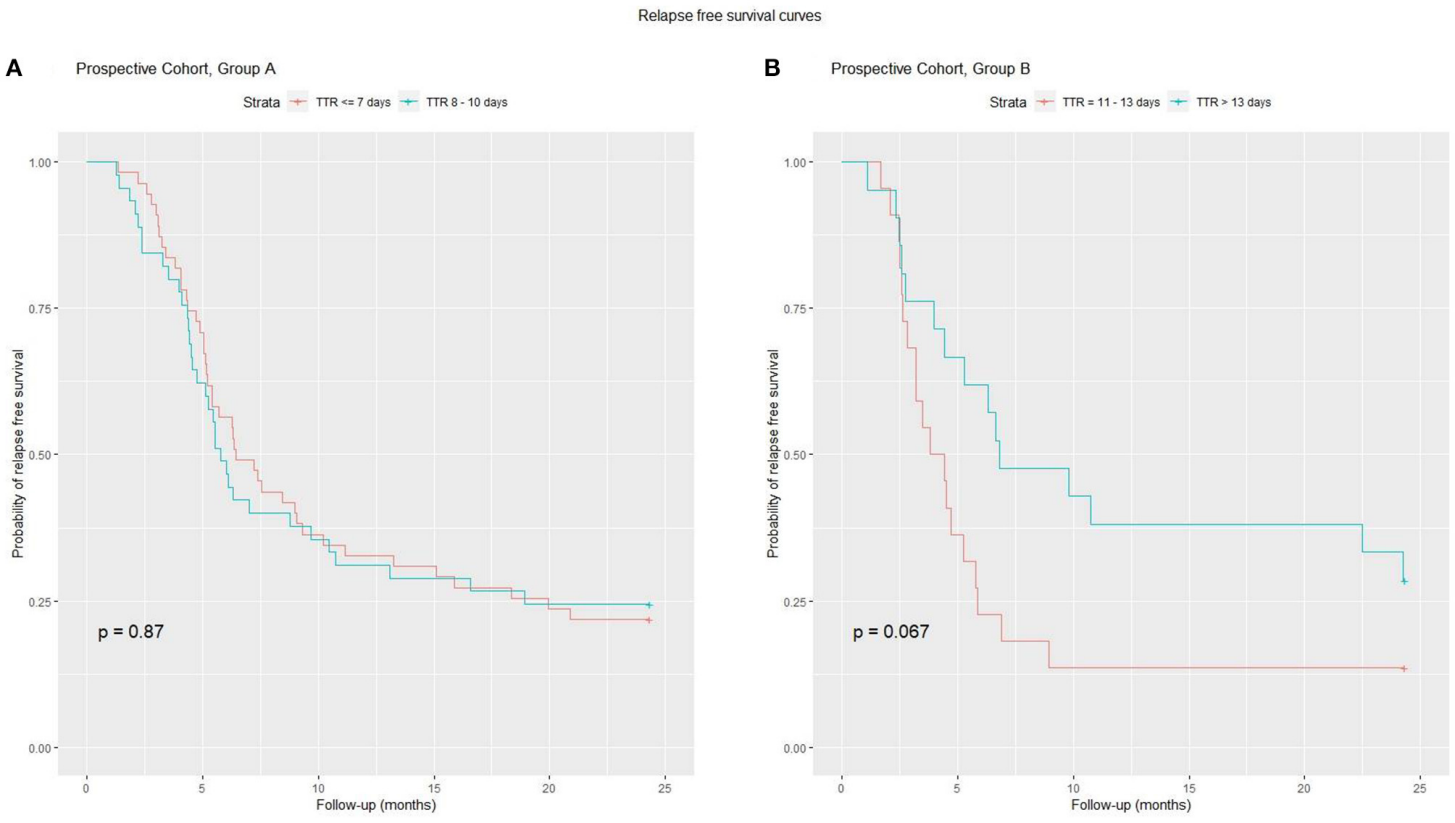
A sub-analysis of TTR and relapse risk was performed on Group A and B. On the left **(A)** for Group A, the relapse-free survival curve did not differ between the two subgroups. On the right **(B)** for Group B patients with higher TTR have a better curve, but the difference is not significant.

In order to exclude the confounding factor of a different induction therapy in the evaluation of TTR, we decided to perform a separate sub-analysis for Group A (induction prednisone dose 1,685 ± 107) and Group B (induction prednisone dose 2,648 ± 426) (Figure 4), categorizing patients as “lower TTR” and “higher TTR” in each group. In both groups, there was no detectable effect of TTR as a negative prognostic factor. When patients from the retrospective cohort were categorized for TTR in the same way as the prospective cohort, data did not show a significant prognostic role for TTR. All total prednisone doses (taken by Group A, Group B, prospective and retrospective cohort) expressed as means ± SD (mg/m^2^), are shown in Figure 3C.

#### Total Induction Prednisone Dose

In the whole population, induction dose was not associated with any of the outcomes (Table 2). The same was seen when the two cohorts were analyzed separately.

#### Laboratory Values at Onset (Table 2)

Total protein values were significantly associated with all outcomes in the entire population. Albumin behaved similarly for three outcomes. A lower total protein or albumin value was associated with higher relapse rate. The behavior was similar in the two cohorts (Supplementary Tables 1, 2). Among other values, only creatinine, proteinuria, and the PrU/CrU ratio showed a consistent association with most outcomes (Table 2). Creatinine showed a paradoxical association: higher values were associated with a better prognosis. As creatinine showed a strong collinearity with age, this association was not analyzed further. The other laboratory values were not consistently associated with the outcomes.

#### Relapse Free Survival Analysis

The last two columns in Table 2 report the results of bivariate analysis for each prognostic factor using relapse free survival as outcome. Both raw *p*-values and adjusted *p*-values are shown. Time to first relapse was always computed from start of therapy, and follow-up was 24 months from start of therapy for all patients.

We chose to use relapse-free survival as outcome for our final multivariate analysis, because it includes information about both relapse rate and time to relapse.

Multivariate analysis was performed using a Cox proportional hazards model, including all the variables with a *p* < 0.1 in the bivariate analysis performed on the whole population. The following variables were excluded because of strong collinearity: albumin with total serum protein, creatinine with age, and PrU/CrU with proteinuria and age. In the final model, age at onset (coefficient = −0.128; *p* = 0.000004) and total serum protein (coefficient = −0.488; *p* = 0.00002) were the only significant independent predictors of relapse free survival (total likelihood ratio = 1.774e-10). Younger patients with lower total serum protein values at onset had a higher risk of earlier and more frequent relapses. None of the other risk factors were independent prognostic factors.

**Figure 5:**
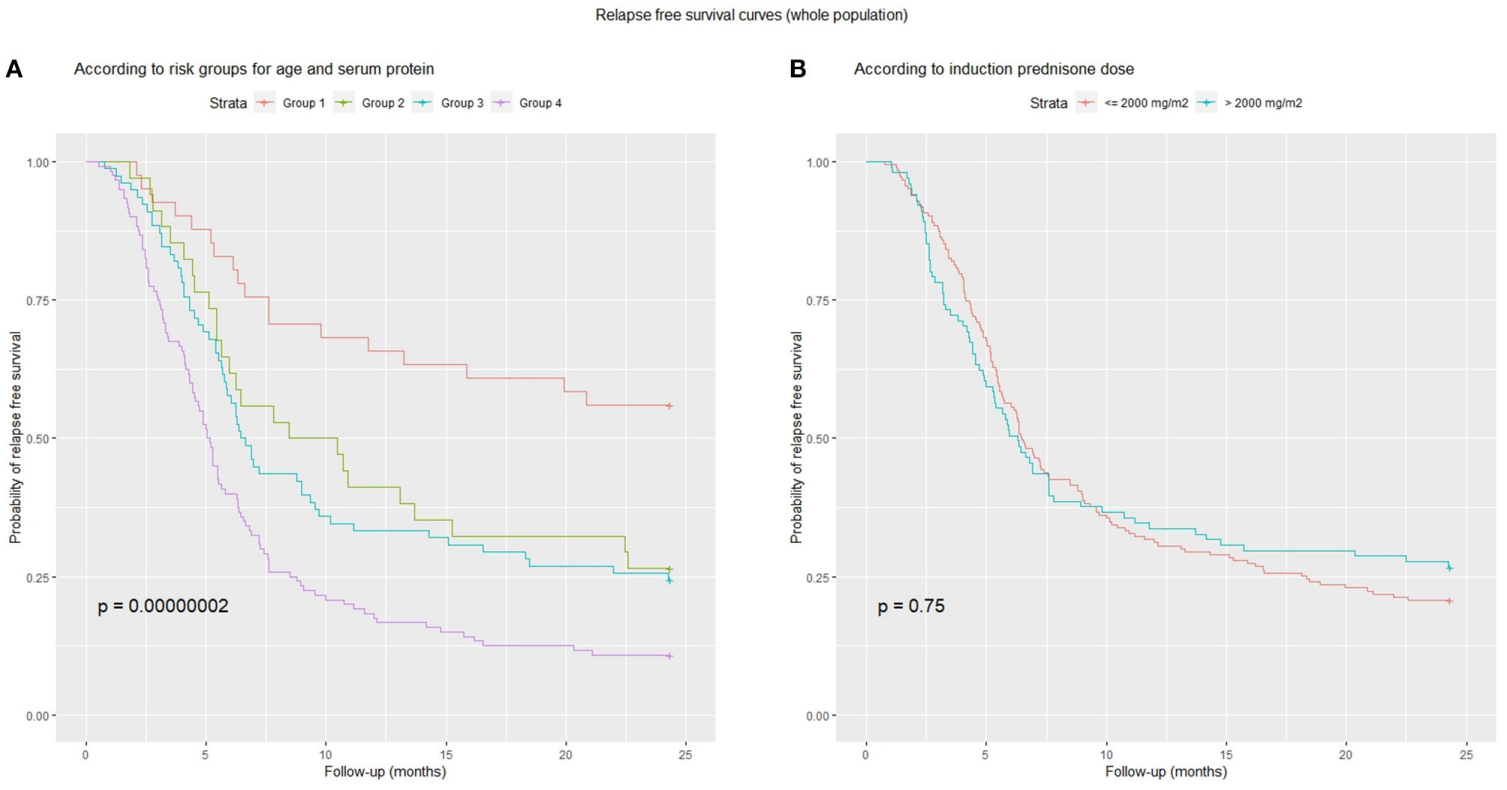
Relapse-free survival curves: **(A)** combined risk groups according to age (cut-off 5.3 years) and serum protein (cut-off 4.2 g/dl): Group 1, low-risk for both; Group 2, high-risk for total proteins only; Group 3, high-risk for age only; Group 4, high-risk for both; **(B)** induction prednisone dose in binary form (threshold 2,000 mg/m^2^).

**Figure 6:**
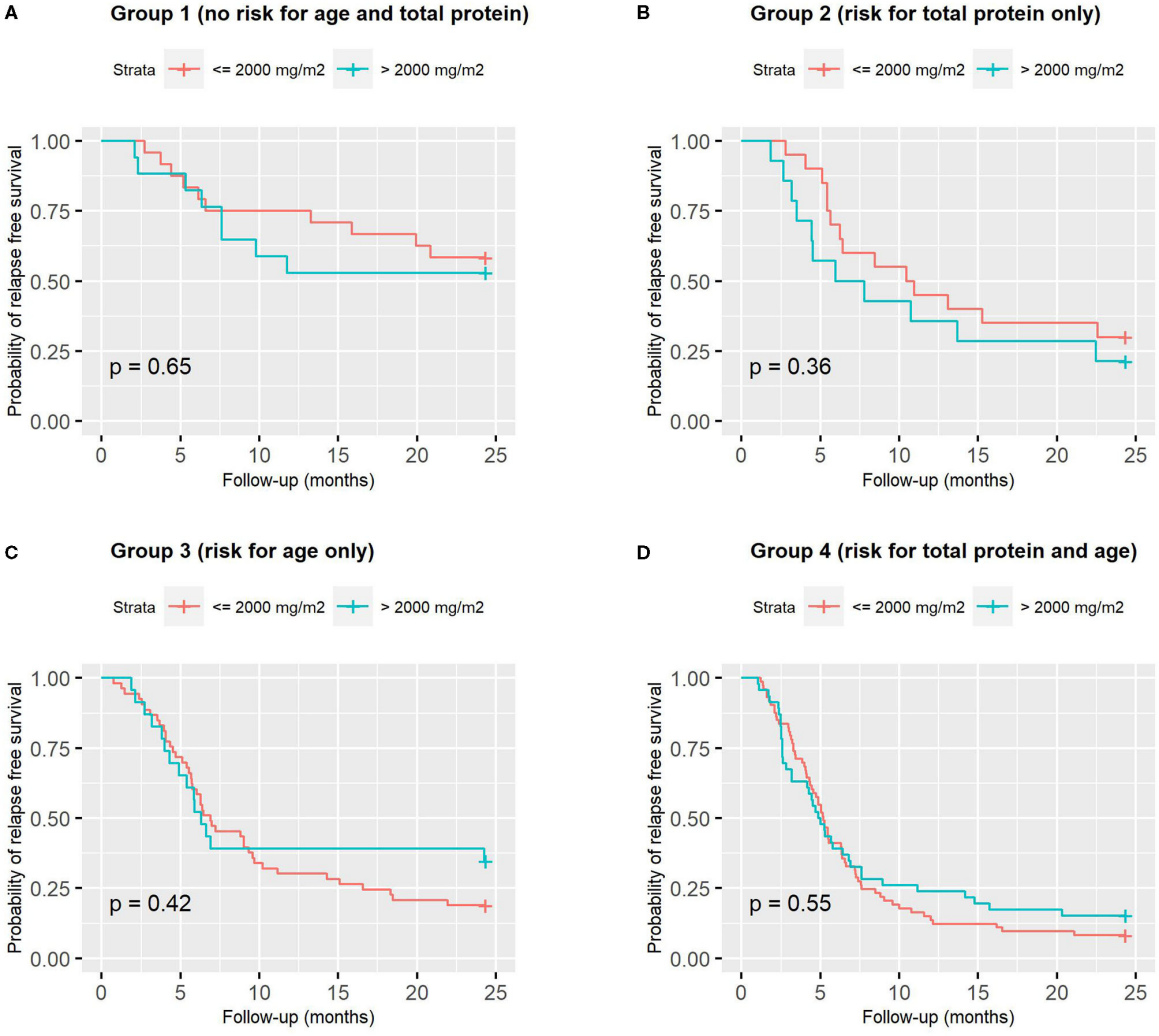
Relapse-free survival curves according to induction prednisone dose in binary form (threshold 2,000 mg/m^2^), analyzed in each of the 4 combined risk groups based on age (cut-off 5.3 years) and serum protein (cut-off 4.2 g/dl): **(A)** Group 1, low-risk for both; **(B)** Group 2, high-risk for total proteins only; **(C)** Group 3, high-risk for age only; **(D)** Group 4, high-risk for both.

A ROC analysis showed that the best cut-offs (Youden's index) for age at onset (AUC = 0.681) and serum protein (AUC = 0.653) were 5.3 years and 4.2 g/dL, respectively. Using these cut-offs, children were categorized as follows: Group 1: not at risk for either total protein or age (*n* = 41, 15%), Group 2: at risk for total protein only (*n* = 34, 12%), Group 3: at risk for age only (*n* = 78, 29%) and Group 4: at risk for both total protein and age (*n* = 120, 44%).This categorization significantly predicted relapse free survival (Figure 5A). Figure 5B shows instead that total induction prednisone dose (threshold 2,000 mg/m^2^) was not significant (*p* = 0.70). Even when analyzing induction prednisone dose in each of the abovementioned risk groups separately, no significant effect on relapse free survival was observed (Figures 6A–D).

These 4 groups were also good predictors of FR-SD prevalence (*p* = 0.007). In Group 1, 24% of patients were FR-SD vs. 54% in Group 4. Groups 2 and 3 showed similar behavior, with 38 and 41% of FR-SD patients, respectively.

## Discussion

This is the first published prospective study to use a steroid regimen adjusted for a prognostic factor. When this study was conceived, the Cochrane review (28) recommended prolonging the total prednisone dose and steroid protocol duration. In accordance with this, we prolonged and diversified our steroid regimen on the basis of TTR ≤ 10 or >10 days. The rationale was to administer a higher daily prednisone dose to children at risk of more frequent relapses or steroid dependence (23–25).

However, we do acknowledge that the original design of the prospective study was not really appropriate, for two important reasons:

1) It was based on the assumption that TTR is a strong negative prognostic factor for relapse. That assumption was based on rather limited evidence from the literature, and was not confirmed in successive studies. Moreover, our same results did not seem to support our initial assumption.
2) The lack of randomization and the association of TTR with different steroid therapy make it very difficult to distinguish between the possible effects of the two variables in our prospective data.

In particular, our results showed that a higher-dose and longer initial steroid therapy for childhood INS, diversified according to TTR, was not associated with a significantly different clinical course. At first glance, this result may appear to mean that treating the patients at higher risk of relapse with higher prednisone doses was successful, having reduced their relapse rate. Unfortunately, that conclusion holds only if the assumption of a strong negative prognostic role of TTR is confirmed.

To draw more reliable conclusions, we decided to expand our data by adding data from a previous retrospective cohort of patients, as described in Methods. At the same time, we performed specific sub analyses of our data aimed at differentiating, as much as possible, between the effect of TTR and the effect of therapy.

As a result of a more in-depth evaluation of our prospective data and a re-evaluation of all the prognostic factors in the whole population of our patients (prospective + retrospective), we found no evidence the TTR can be considered a major prognostic factor of relapse (Figure 4, Supplementary Figures 1, 2). Similarly, no detectable effect of different steroid doses (in the range of those tested) was found.

### Relapse Rate

Relapse rate at 24 months, time to relapse, the number of relapses per patient and the percentage of FR+SD subjects did not differ significantly either between Groups A and B, or between the prospective and retrospective cohorts, where no TTR-based categorization was applied.

Our data confirm the results of three RCTs, published when our study was ongoing, which showed that relapse rate was not modified by initial steroid regimen. In 2013, Teeninga demonstrated that extending initial prednisone treatment without increasing cumulative dose did not benefit clinical outcome (29). In 2015, Sinha et al. (30) and Yoshikawa et al. (31), comparing a 3-month (2,792 ± 287 mg/m^2^) vs. a 6-month regimen (3,530 ± 399 mg/m^2^), and a 2- vs. a 6-month regimen (2,240 vs. 3,885 mg/m^2^), respectively, found no differences in relapse rate or the number of FR subjects (32). These findings were confirmed both by the authors of the 2015 Cochrane review (33) and the results of a recent RCT (34).

In particular, in our study, considering the total induction dose in binary form in the whole population, low-dose (<2,000 mg/m^2^) was as effective as high-dose (>2,000 mg/m^2^) in terms of relapse free survival (Figure 5B), and the same was true considering each risk group (based on age and total protein) separately (Figures 6A–D). Sinha obtained similar results in his RCT (30), comparing 3- vs. 6-month regimens.

As TTR was apparently associated with some differences in laboratory data at diagnosis, we suggest that it could be perhaps considered as a consequence of delayed diagnosis and more serious renal involvement at onset, rather than as a prognostic factor for a subsequent higher relapse rate.

On the contrary, both age and total serum protein concentrations at onset (but also proteinuria and uPr/uCr) were shown to be consistent predictors of relapse, independently of TTR and steroid induction doses.

Indeed, a more immature immunological status in younger patients could be related to a higher frequency of relapses and steroid dependency, as reported by many studies (18–21). Concurrently, a massive proteinuria causing a greater reduction of total serum protein concentrations, could be, at onset, a clinical prognostic sign of subsequent frequent relapses.

#### Cumulative Prednisone Doses

Another aspect we wanted to investigate in our study was the possible role of higher steroid dose at onset in reducing the need for steroids after remission. Our data definitely do not support that hypothesis, as shown by the evaluation of the mean cumulative doses taken from the end of the induction to 24 months (Figure 3A). This result cannot be attributed to a different use of steroid sparing agents.

### Prognostic Factors

As previously discussed, our data did not confirm a major prognostic role for TTR, contrary to published studies (20, 22, 35–39).

Conversely, age at onset and total serum protein were significant and strong prognostic factors, associated with all the outcomes. A correlation between age at onset and risk of relapse has been shown by many authors (15, 16, 18, 30, 40, 41), but not by others (37). In 2003, an RCT comparing long vs. short-course prednisolone regimens (42) showed that younger children were more susceptible to relapse and benefitted from the long alternate-day regimen. In 2015, Sinha's *post-hoc* analysis (30) showed that age ≤ 3 years was associated with an increased relapse risk, but the number of FR children was not reduced by prolonged steroid therapy. The relapse rate and number of FR + SD subjects was not lower in younger children treated with higher prednisone doses.

In a multivariate analysis performed for relapse free survival in our whole population, total serum protein and age at onset were confirmed as the only independent prognostic factors. Based on the ROC analysis cut-offs (5.3 years and 4.2 g/dL), it was possible to categorize children into groups with different relapse risks (Figure 5A).

Nevertheless, a small percentage of younger subjects with lower serum protein never relapsed. Other factors are probably involved. Recent research has focused on the impact of genetic polymorphisms on glucocorticoid response (43–45).

### Limitations

The non-optimal design of the original prospective study motivated us to add a retrospective cohort of patients to perform a more reliable analysis. Of course, that can be in itself a cause of bias and of difficulties in interpretation of the results. However, we believe that that procedure allowed us to comprehensively analyse a large population of patients, especially for prognostic factor analysis. Our analysis shows that data from the two cohorts are comparable enough, and that our main conclusions are reliable and consistent across the two individual cohorts.

The choice of 10 days as a cut-off value for TTR was based on our retrospective study. In the prospective study, the distribution of TTR was shifted toward the left, with a median of 8 days, resulting in an unbalanced number of patients (100 vs. 43). Moreover, we did not confirm TTR as a significant predictive factor, as also suggested by recent studies.

Further limitations include the number of dropouts (20) for non-adherence.

## Conclusions

Steroid doses, adjusted for TTR, did not modify either the relapse rate or the number of FR + SD children at 24 months. However, a more refined analysis of our data showed that TTR cannot be considered a major prognostic factor of relapse, differently from what was reported by some other authors.

Conversely, younger age and low total serum protein values at onset were reliable prognostic factors for relapse and steroid dependence, with no apparent effects of higher prednisone regimens at onset. According to our data, a 4-week steroid induction regimen does not require higher cumulative doses throughout 24 months of follow-up, and thus represents a valid option for pediatric INS. However, a deeper understanding of the pathogenetic mechanisms involved in INS will help us detect subjects at higher risk of relapse and choose new therapeutic options (46–50).

## Data Availability Statement

The raw data supporting the conclusions of this article will be made available by the authors, without undue reservation.

## Ethics Statement

The studies involving human participants were reviewed and approved by Comitato Etico Indipendente di Area Vasta Emilia Centro (CE-AVEC) della Regione Emilia-Romagna IRCCS Azienda Ospedaliero-Universitaria di Bologna, Policlinico S.Orsola-Malpighi. Written informed consent to participate in this study was provided by the participants' legal guardian/next of kin.

## Author Contributions

AP, AE, GMo conceptualized and designed the study, coordinated and supervised data collection, drafted the initial manuscript, reviewed, and revised the manuscript. GP carried out the initial analyses, drafted the initial manuscript, reviewed, and revised the manuscript. CB, LC, CC, GG, LG, MG, CL, CM, SM, AM, MM, FM, GMe, EM, WM, and PR collected data and reviewed and revised the manuscript. All authors approved the final manuscript as submitted and agree to be accountable for all aspects of the work.

## Members of The Nefrokid Study Group (Italy)

Andrea Pasini, Francesca Mencarelli, Chiara De Mutiis, Claudio La Scola, Cristina Bertulli: Nephrology and Dialysis Unit, Department of Pediatrics, IRCCS Azienda Ospedaliero-Universitaria di Bologna; Elena Monti: Specialty School of Paediatrics - Alma Mater Studiorum, Università di Bologna, Italy; Gabriella Aceto, Giovanni Messina, Laura Spagnoletta, Mario Giordano, Tommaso De Palo: Nephrology Division, Giovanni XXIII Children's Hospital, Bari; Giovanni Montini, Alberto Edefonti, Luciana Ghio, Gianluigi Ardissino, Antonio Mastrangelo, William Morello, Elena Groppali, Marta Lepore: Pediatric Nephrology and Dialysis Unit, Fondazione Ca' Granda IRCCS Ospedale Maggiore Policlinico, Milan; Silvio Maringhini, Vitalba Azzolina, Ciro Corrado: Pediatric Nephrology Unit, Children's Hospital G. Di Cristina, A.R.N.A.S. Civico, Palermo; Carmelo Fede, Roberto Chimenz, Giovanni Conti: Pediatric Nephrology and Dialysis, AOU G.Martino, Messina; Paola Romagnani, Marco Materassi, Fiammetta Ravaglia: Nephrology and Dialysis Unit, Meyer Children's Hospital, Florence; Maria D'Agostino: Pediatric Unit, S.Giovanni XXIII Hospital, Bergam; Sante Cantatore: Department of Pediatrics, Azienda Ospedaliera - University of Modena, Modena; Anita Ammenti, Claudio Ruberto: Department of Pediatrics, University of Parma, Parma; Chiara Gualeni: Pediatric Unit, Children's Hospital, Brescia; Elena Cama: Department of Pediatrics and Neonatology, Desenzano del Garda; Mariotti Paola: Pediatric Unit, San Jacopo Hospital, Pistoia; Amata Negri: Pediatric Unit, Filippo Del Ponte Hospital, Varese; Alberto Bettinelli: Pediatric Unit, San Leopoldo Mandic Hospital, Merate; Gianluca Vergine, Elisa Ravaioli, Alessandra Lavacchini: Pediatric Unit, Ospedale degli Infermi, Rimini; Alessandra Dozza: Pediatric Unit, Ospedale Maggiore, Bologna; Angela Simoni: Pediatric Unit, Ramazzini Hospital, Carpi; Marina Piepoli: Pediatric Unit, Guglielmo da Saliceto Hospital, Piacenza; Felice Sica: Pediatric Unit, AOU Ospedali Riuniti, Foggia; Gabriele Ripanti: Pediatric Unit, San Salvatore Hospital, Pesaro; Marina Milani: Pediatric Unit, Fondazione MBBM, S. Gerardo Hospital, Monza; Paola Tommasi: Pediatric Unit, Vittore Buzzi Hospital, Milan; Carla Romanello: Pediatric Unit, S. Maria della Misericordia Hospital, Udine; Manuela Pasini: Pediatric Unit, Maurizio Bufalini Hospital, Cesena; Paola Mastinu: Pediatric Unit, S.Chiara Hospital, Trento; Laura Luti: Pediatric Unit, AOU Pisa; Antonella Amendolea: Pediatric Unit, Cecina; Silvia Manfredi: Pediatric Unit, Massa Carrara; Fabrizio Pugliese: Pediatric Emergency Department, Salesi Children's Hospital, University of Marche; Emanuela Lanfranchi: Pediatric Unit, Fermo; Maria Principi: Pediatric Unit, Macerata; Agrippino Reciputo: Pediatric Unit, Cinisello Balsamo; Marialuisa Casciana: Pediatric Unit, C. Poma Hospital, Mantova; Antonio Pellegatta: Pediatric Unit, Busto Arsizio; Fiorella Russo: Pediatric Unit, Desio; Nicola Altamura: Pediatric Unit, Sesto San Giovanni; Lorena Ruzza: Pediatric Unit, San Carlo Hospital, Milan; Stefano Sardini: Pediatric Unit, Asola; Ines L'Erario: Pediatric Unit, Burlo Garofalo Hospital, Trieste; Antonella Crisafi: Pediatric Unit, Santa Maria Nuova Hospital, Reggio Emilia; Andrea Zucchini: Pediatric Unit, Faenza; Laura Serra: Pediatric Unit, Santa Maria della Scaletta Hospital, Imola; Peter Bertamini: Pediatric Unit, Santa Maria del Carmine Hospital, Rovereto; Rita Bini: Pediatric Unit, Grosseto; Patrizia Cortesi: Pediatric Unit, Pescia; Caterina Balducci: Pediatric Unit, Prato; Francesca Simoni: Pediatric Unit, Pianadi Lucca Hospital, Lucca; Patrizia Fonduli, Franca Paola Zurrida: Pediatric Unit, Brotzu Hospital, Cagliari; Laura De Petris: Pediatric Unit, Mazzoni Hospital, Ascoli Piceno; Vinicio Goj: Pediatric Unit, Fatebenefratelli Hospital, Milan; Gian Luigi Marsiglia: Pediatric Unit, IRCCS Policlinico S Matteo, Pavia; Patrizia Caruso: Pediatric Unit, Cremona; Filippo Salvini: Pediatric Unit, S. Paolo Hospital, Milan; Paola Perotti: Pediatric Unit, Voghera; Anna Bussolini: Pediatric Unit, Tradate; Stefano Poli, Barbara Balduzzi: Pediatric Unit, Esine; Barbara Roman: Pediatric Unit, Vimercate; Sergio Mariani, Laura Cafarelli: Pediatric Unit, Saronno; Vittorio Venturoli: Pediatric Unit, Morgagni-Pierantoni Hospital, Forlì; Andrea Corsini Pediatric Unit, Bentivoglio; Luca Casadio: Pediatric Unit, Ravenna.

## Conflict of Interest

The authors declare that the research was conducted in the absence of any commercial or financial relationships that could be construed as a potential conflict of interest.

## Abbreviations

INS: idiopathic nephrotic syndrome
TTR: time to remission
uPr/uCr: urine protein/creatinine
NR: non-relapser
IR: infrequent relapse
FR: frequent relapser
SD: steroid-dependent
SR: steroid-resistant
SDS: systolic and diastolic blood pressure
SS: steroid-sensitive.
