# Supplementary material for: Childhood Idiopathic Nephrotic Syndrome: Does the Initial Steroid Treatment Modify the Outcome? A Multicentre, Prospective Cohort Study
Source: Front Pediatr. 2021 Jul 8;9:627636. doi: 10.3389/fped.2021.627636 (PMC8295604; doi:10.3389/fped.2021.627636)
Supplement: Supplementary file 1 [file Data_Sheet_1.doc]

Supplementary Material


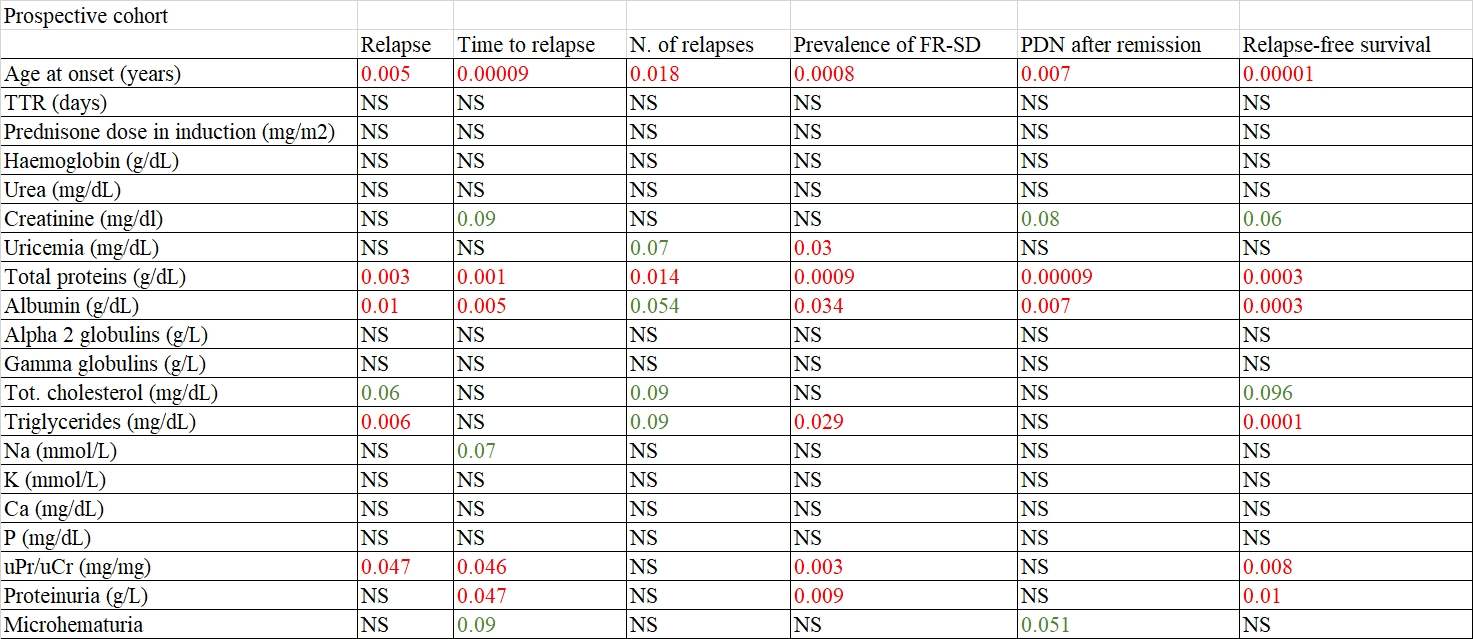


**Supplementary Table 1.** Prognostic factors evaluated in the prospective cohort, according to the different outcomes. p values are in red if < 0.05, in green if >= 0.05 and < 0.10. All p values > 0.10 are given as NS.

**Supplementary Table 2.** Prognostic factors evaluated in the retrospective cohort, according to the different outcomes. p values are in red if < 0.05, in green if >= 0.05 and < 0.10. All p values > 0.10 are given as NS.


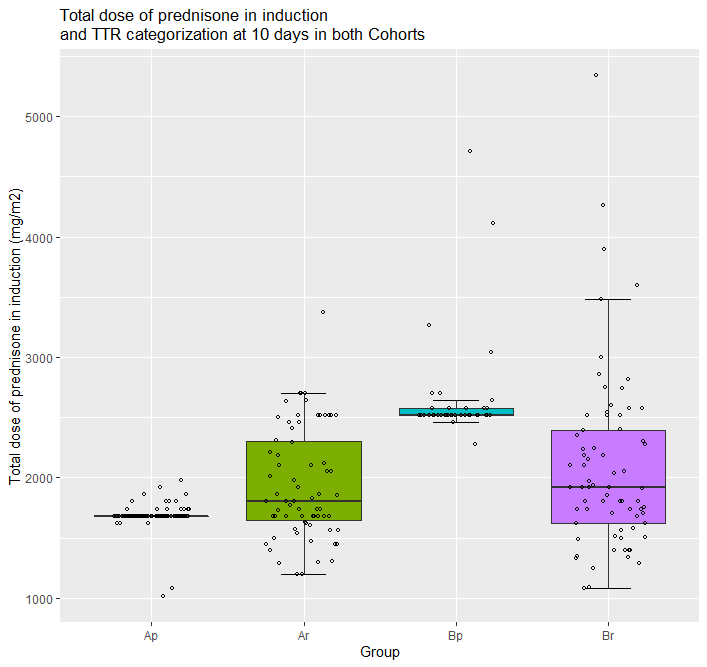


**Supplementary Figure 1.** *TTR groups and total prednisone dose in induction.*

For this further sub-analysis, we categorized patients in the retrospective cohort according to TTR values using the same threshold as in the prospective study: <= 10 days, > 10 days. Ap and Bp are the two groups in the prospective cohort. Ar and Br are the corresponding groups in the retrospective cohort. Prednisone dose in induction was determined by protocol, and therefore very strict (with a few outliers) in Groups Ap and Bp, while in Groups Ar and Br it was essentially similar, and intermediate between the two cumulative doses of the prospective cohort. This sets the context for a more refined analysis of the relationship between TTR categorization, induction dose and clinical course (see next figure).


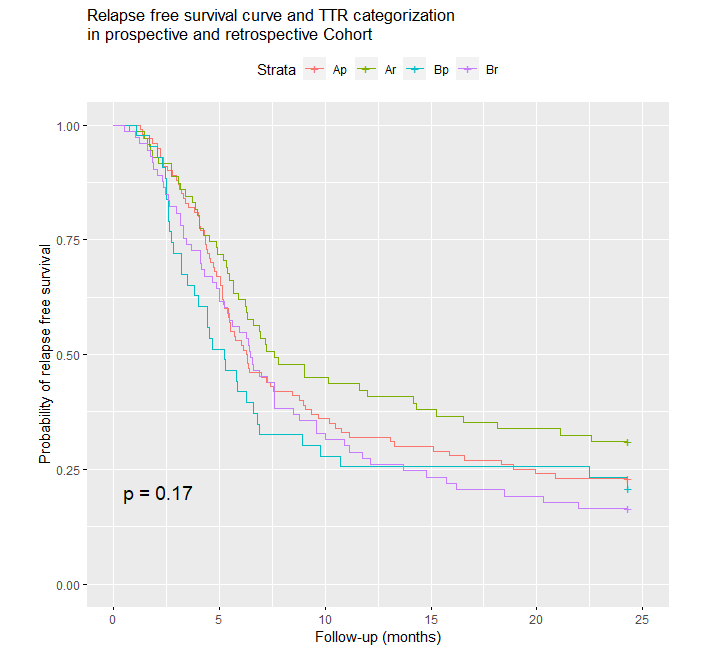


**Supplementary Figure 2.** *TTR groups and relapse free survival.*

The four groups defined above, and the corresponding relapse free survival curves. No statistical significance is observed.
